# Neuroprotection from Excitotoxic Injury by Local Administration of Lipid Emulsion into the Brain of Rats

**DOI:** 10.3390/ijms21082706

**Published:** 2020-04-14

**Authors:** Motomasa Tanioka, Wyun Kon Park, Insop Shim, Kyeongmin Kim, Songyeon Choi, Un Jeng Kim, Kyung Hee Lee, Seong-Karp Hong, Bae Hwan Lee

**Affiliations:** 1 Department of Physiology, Yonsei University College of Medicine, Seoul 03722, Korea; hpark@yuhs.ac (M.T.); kkm0814@yuhs.ac (K.K.); sychoi@yuhs.ac (S.C.); mignon@yuhs.ac (U.J.K.); 2 Brain Korea 21 PLUS Project for Medical Science, Yonsei University College of Medicine, Seoul 03722, Korea; 3 Department of Anesthesiology and Pain Medicine, Anesthesia and Pain Research Institute, Yonsei University College of Medicine, Seoul 03722, Korea; wkp7ark@yuhs.ac; 4 Department of Physiology, School of Medicine, Kyung Hee University, Seoul 02447, Korea; ishim@khu.ac.kr; 5 Department of Dental Hygiene, Division of Health Science, Dongseo University, Busan 47011, Korea; kyhee@gdsu.dongseo.ac.kr; 6 Division of Biomedical Engineering, Mokwon University, Daejeon 35349, Korea; karp@yuhs.ac

**Keywords:** neuroprotection, hippocampus, lipid emulsion, intralipid, excitotoxicity, kainic acid

## Abstract

Lipid emulsion was recently shown to attenuate cell death caused by excitotoxic conditions in the heart. There are key similarities between neurons and cardiomyocytes, such as excitability and conductibility, which yield vulnerability to excitotoxic conditions. However, systematic investigations on the protective effects of lipid emulsion in the central nervous system are still lacking. This study aimed to determine the neuroprotective effects of lipid emulsion in an in vivo rat model of kainic acid-induced excitotoxicity through intrahippocampal microinjections. Kainic acid and/or lipid emulsion-injected rats were subjected to the passive avoidance test and elevated plus maze for behavioral assessment. Rats were sacrificed at 24 h and 72 h after kainic acid injections for molecular study, including immunoblotting and qPCR. Brains were also cryosectioned for morphological analysis through cresyl violet staining and Fluorojade-C staining. Anxiety and memory functions were significantly preserved in 1% lipid emulsion-treated rats. Lipid emulsion was dose-dependent on the protein expression of β-catenin and the phosphorylation of GSK3-β and Akt. Wnt1 mRNA expression was elevated in lipid emulsion-treated rats compared to the vehicle. Neurodegeneration was significantly reduced mainly in the CA1 region with increased cell survival. Our results suggest that lipid emulsion has neuroprotective effects against excitotoxic conditions in the brain and may provide new insight into its potential therapeutic utility.

## 1. Introduction

Excitotoxicity is considered a major mechanism underscoring neurodegenerative disorders involving functional loss and death of neurons in the central nervous system (CNS) [1,2]. Excitotoxic conditions have been implicated in acute and chronic neurodegenerative diseases, such as Alzheimer’s disease, Parkinson’s disease, Huntington’s disease, and epilepsy [3]. In animal models of excitotoxic neurodegeneration, chemical convulsants such as kainic acid (KA) have been utilized to mimic pathological conditions observed in patients [4,5]. KA is a neurotoxic analogue of glutamate that binds to kainate receptors, resulting in overstimulation of neurons at high doses [6]. KA-induced excitotoxicity in rodents results in deficient cognitive functions [7], elevated anxiety levels [8], and disruptive morphological changes in different areas of the brain [9,10]. In particular, the hippocampus has been established as a site of damage following KA administration, which elicits cognitive dysfunction [11,12]. Numerous therapeutic approaches have been investigated to terminate convulsive seizures induced by KA; however, the pursuit of appropriate remedies against neural damage induced by excitotoxic conditions is an ongoing endeavor.

Previous studies reported that the expression of the Dickkopf-related protein 1 (Dkk-1), an antagonist of the canonical Wnt signaling pathway that promotes GSK3-β activity, was elevated in biopsies of patients [13] and animal models [14,15] undergoing neurodegeneration. Proteasomes subsequently degrade β-catenin, a downstream survival marker of GSK3-β, through ubiquitination, which often leads to cell death. Wnt is a canonical lipid-modified signaling glycoprotein that regulates the phosphorylation of GSK3-β [16]. The brain tissue of KA-administered rats expressed higher antagonistic activity of Wnt [17], indicating that the Wnt signaling pathway may have an important role in neurodegenerative excitotoxicity of the brain.

In 1962, lipid emulsion (LE) was approved for clinical use as a component of parenteral nutrition. Composition of lipid emulsion (Intralipid™ 20%, Fresenius Kabi, Uppsala, Sweden) is 20% soybean oil, 1.2% egg yolk phospholipids, 2.25% glycerin, and water for injection. The major component fatty acids of soybean oil are linoleic (44%–62%), oleic (19%–30%), palmitic (7%–14%), linolenic (4%–11%), and stearic (1.4%–5.5%). Then in 1998, Weinburg et. al. [18] proposed a different role of LE as a resuscitation method for treating local anesthetic systemic toxicity (LAST). Recent clinical case reports utilized LE to rescue patients from LAST and promote recovery from cardiac toxicity induced by lidocaine [19], ropivacaine [20], or bupivacaine [21]. The protective mechanisms underscoring LE treatment include the phosphorylation of protein kinase B (Akt) [22] and glycogen synthase kinase-3β (GSK3-β), hence promoting cell survival [23,24]. Extensive researches like the aforementioned studies were conducted in cardiac cells over the past decades, but systematic investigations on the protective effects of LE in the CNS are still lacking. There are key similarities between neurons and cardiomyocytes such as excitability and conductibility, which yield vulnerability to excitotoxic conditions. Cardioprotective properties that have been reported from studies are sometimes diagnostic tools or therapeutic targets for neuroprotection [25,26]. Similitude of these organs shed light on speculations of the protective effects of LE in the brain. Despite the accumulating conjectures about the effect of LE on neuroprotection, studies have not yet assessed its protective effects in the brain. Our study aimed to elucidate the protective effects of LE when administered directly into the excitotoxic brain

Therefore, in this study, we investigated the neuroprotective effects of LE in an in vivo rat model of KA-induced excitotoxicity. We examined neuroprotection by measuring the preservation of hippocampal function through behavioral tests after acute damage of hippocampal neurons. We also assessed the changes in mRNA expression of *Wnt1*, *Wnt3*, and *Wnt5a,* which are representative genes of canonical and non-canonical Wnt signaling pathways to further investigate the signaling affected by neuroprotection. Additionally, we report changes in protein expression levels of downstream markers of the canonical Wnt signaling pathway in relation to cell survival. We also provide data on neurodegeneration and morphological changes in the hippocampus. Based on behavioral studies, molecular analysis, and morphological examinations, we propose that LE provides neuroprotection against excitotoxicity in the brain.

## 2. Results

### 2.1. Seizure and Survival

**Figure 1:**
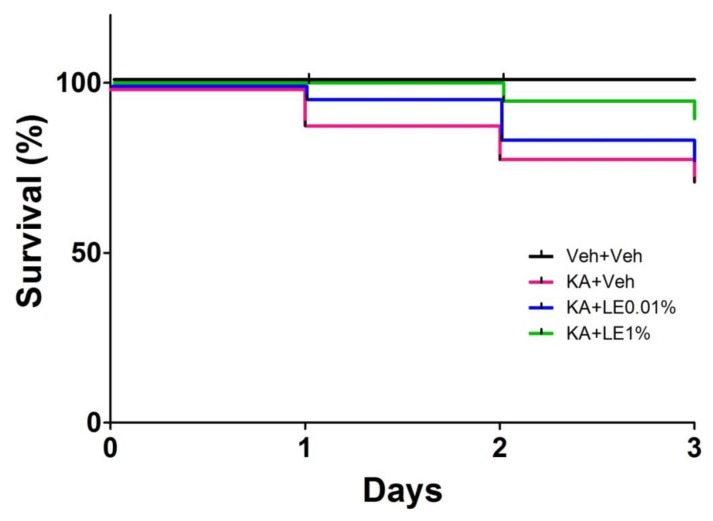
Seizure severity after kainic acid (KA) injection and survival rate for each experimental group. Survival rate of experimental animals up to 3 days post-kainic acid injection. (*n* = 65 per group, *p* = 0.0024 for Veh + Veh vs. KA + Veh, *p* = 0.6063 for KA + Veh vs. KA + LE 0.01%, *p* = 0.0772 for KA + Veh vs. KA + LE 1%; survival analyzed by log-rank [Mantel-Cox] test).

Seizure severity was observed in groups administered with KA. Only rodents that experienced stage 3 seizure severity or higher were used in our experiments; this accounted for approximately 83% (183/220) of KA-administered rats (Table 1). 37/220 rats that have experienced seizure level 2 (facial clonus) or less have been excluded from the study due to the inconsistency in hippocampal damage severity (Table 1). Although KA-injected rats in all groups were administered with an identical dose of KA, there were phenotypic differences in individual seizure severity. The KA + Veh group exhibited a significantly lower survival rate (47/65) than that of the Veh + Veh group (65/65). The impact of LE on survival was not significant but approached a trend for significance (P = 0.0772 for KA + Veh vs. KA + LE 1%; Figure 1, Table 2) by 3 days post-KA injection.

### 2.2. Memory Retention in Behavioral Tests

**Figure 2:**
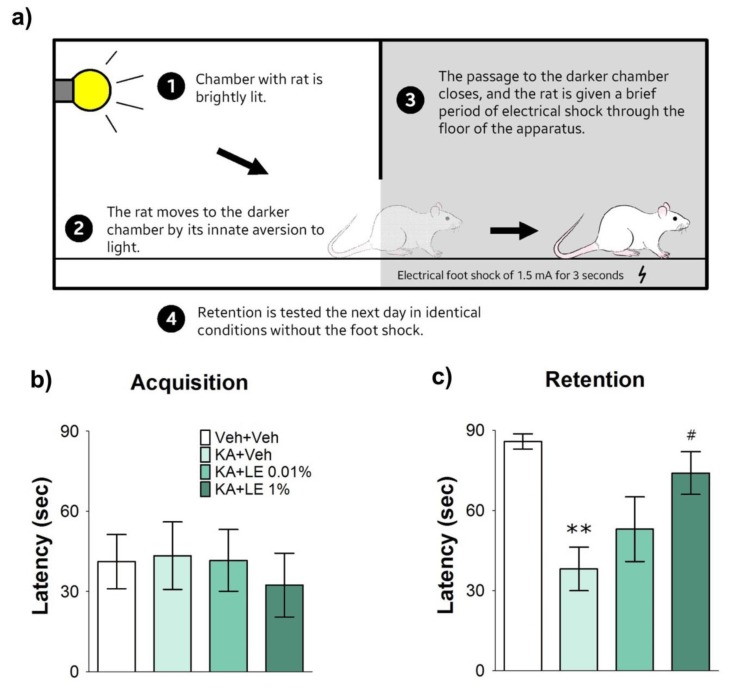
Illustration of the passive avoidance test and results 4 days after kainic acid (KA) and repetitive lipid emulsion (LE) injection. (**a**) A schematic drawing describing the procedures of the single-trial passive avoidance test. The behavioral test consisted of habituation, acquisition, and retention trials at 2, 3, and 4 days after kainic acid injection, respectively. (**b**) Measurements of the stepover latency during the acquisition trials (initial latency). There were no noticeable differences between all experimental groups. (**c**) The stepover latency measured during the retention trial (retention latency). Significant differences in retention latency were recorded in the Veh + Veh, and KA + 1% groups; (**b**–**c**) Data are presented as mean ± standard error of the mean (SEM); *n* = 8 for each group; **p < 0.01 vs. Veh + Veh, #*p* < 0.05 vs. KA + Veh, one-way analysis of variance (ANOVA) followed by Tukey’s multiple comparison test.

Passive avoidance test is a behavioral test that examines learning and memory (Figure 2a). Rodents are fear-conditioned via electrical foot shocks to counteract movement into a favorable environment. Unimpaired rats do not move into the darker chamber, as they have learned that a foot shock is the consequence. However, pathological rats that fail to learn the adverse consequences move into the darker chamber, regardless of conditioning [27].

There were no significant differences in acquisition latency among groups (Figure 2b). The KA + Veh group experienced similar deficits in learning, whereas their retention latency was significantly shorter than that of the Veh + Veh group (*p* < 0.01; Figure 2c). Failure to form new memories is associated with deficient hippocampal activity [27,28]. Rats that experienced acute hippocampal damage were more reactive to light and hyperactive when the chamber lit up. Four rats did not cross over to the darker chamber for the entire session, indicating preservation of hippocampal memory function. The KA + LE 0.01% group was hesitant to enter the darker chamber, but no significant differences in retention latency were observed between the KA + LE 0.01% and KA + Veh groups. The average difference between KA + Veh and Veh + Veh was 47 s, KA + Veh and KA + LE 0.01% 14 s, and KA + Veh and KA + LE 1% 35 s (*n* = 8).

### 2.3. Decreased Anxiety via CA1 Protection

**Figure 3:**
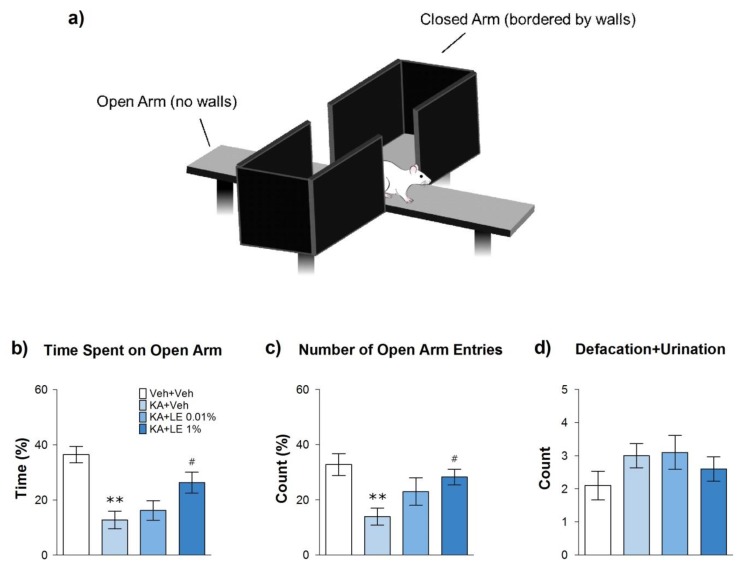
Illustration of the elevated plus maze and results 4 days after kainic acid (KA) and repetitive lipid emulsion (LE) injection. (**a**) A schematic drawing of the elevated plus maze. The behavioral test was conducted 4 days after kainic acid injection. (**b**) Measurement of the time spent in the open arms compared to the total duration. The KA + LE 1% group spent significantly more time in the open arms compared to the KA + Veh group. (**c**) The number of entries into the open arm compared to the total entries into all arms. The KA + LE 1% group entered the open arms significantly more than did the KA + Veh group. (**d**) Defecation and urination count for each experimental group. Data are presented as mean ± standard error of the mean (SEM); *n* = 10 for each group; **p < 0.01 vs. Veh + Veh, #*p* < 0.05 vs. KA + Veh, one-way analysis of variance (ANOVA) followed by Tukey’s multiple comparison test.

Elevated plus maze is a behavioral test that measures anxiety in experimental animals [29]. The maze comprises four arms, two of which are bordered by walls and two of which remain open, in which rats are at risk of falling off the platform (Figure 3a). Rodents have an innate aversion towards open and brighter environments, but they are also curious animals that actively explore novel areas to learn about their environment [30]. KA is known to be anxiogenic in rats by inducing hippocampal damage [31]. Rats deficient in CA1 neurons of the hippocampus tend to be less exploratory and remain stagnant inside the closed arms of the maze [32].

The KA + Veh group were less mobile and spent significantly less time in the open arms compared to the Veh + Veh group (Figure 3b). The KA + LE 1% group demonstrated a significantly longer duration of exploratory behavior in the open arms and was noticeably more active during the behavioral test compared to the KA + Veh group (Figure 3c). Four animals in the KA + LE 0.01% group displayed similar results as those in the KA + LE 1% group (*n* = 10), but the difference in exploratory activity was not statistically significant when compared to the KA + Veh group. Anxiety behavior was inconsistent between individuals. In addition, defecation and urination in the KA + Veh group were more frequent, but there were no significant differences among groups (Figure 3d).

### 2.4. LE alleviates Damage in CA1

**Figure 4:**
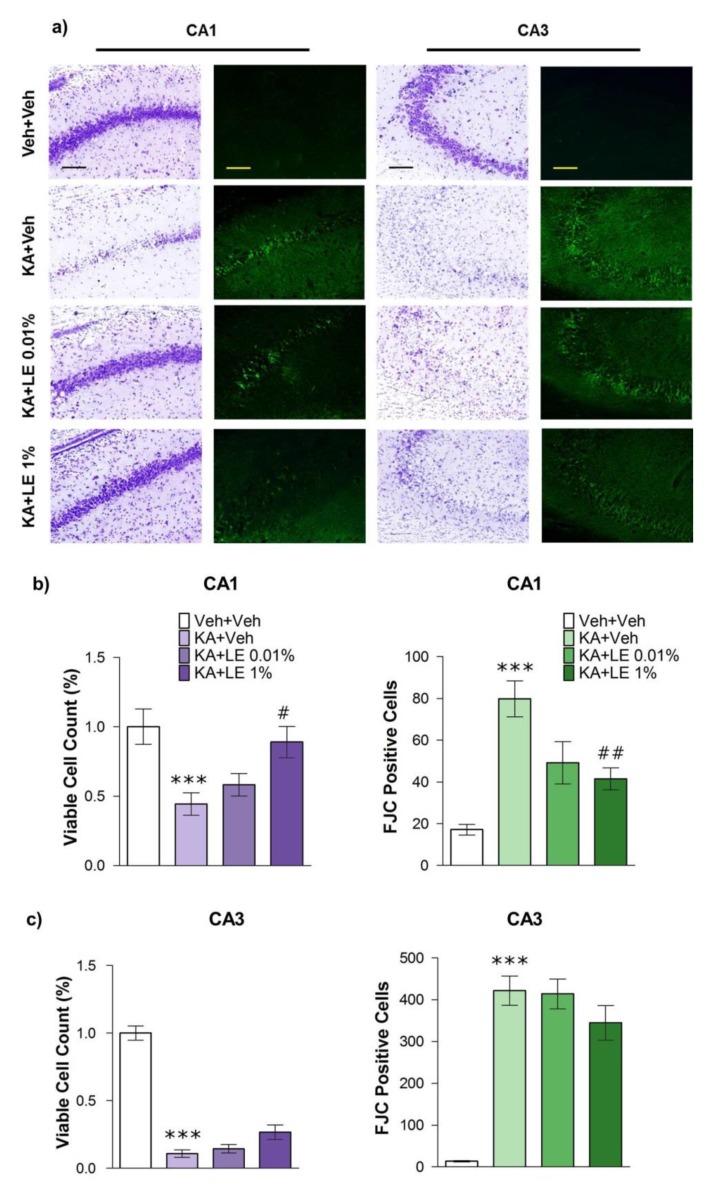
Effects of lipid emulsion on the morphology of hippocampal neurons 72 h after kainic acid (KA) and repetitive lipid emulsion injection (3 times). (**a**) Cresyl violet (Columns 1 and 3) and Fluoro-Jade C (FJC) (Columns 2 and 4) staining for each group in the hippocampal CA1 and CA3 regions. Scale bars = 200 μm. (**b**) Quantification of viable cells compared to Veh + Veh group in the CA1 of the hippocampus. The number of viable cells in the KA + Veh group was significantly lower compared to the Veh + Veh group, while the number of viable cells in the KA + LE 1% group was significantly higher compared to the KA + Veh group (left). Neurodegeneration in the KA + Veh group was significantly increased compared to that in the Veh + Veh group. FJC-positive cells were significantly decreased in the KA + LE 1% group compared to that in the KA + Veh group (right). (**c**) Quantification of viable cells compared to Veh + Veh group in the CA3 region of the hippocampus. The KA + Veh group exhibited a significant decrease in the number of viable cells (left) and significant increase in the measurement of neurodegeneration compared to the Veh + Veh group (right). No significant differences were observed in LE-treated groups compared to the KA + Veh group. Data are presented as mean ± standard error of mean (SEM); *n* = 12 for each group; *** *p* < 0.001 vs Veh + Veh, # *p* < 0.05, ## *p* < 0.01 vs. KA + Veh, one-way analysis of variance (ANOVA) followed by Tukey’s multiple comparison test.

Morphological changes in the hippocampus at 72 h after KA administration have been reported in numerous studies [17,33]. In particular, CA1 and CA3 regions of the hippocampus are prone to damage [34,35], which may lead to severe impairments in cognitive functions. Therapeutic approaches to prevent excitotoxic cell death in hippocampal regions have been extensively investigated. We examined cell viability in CA1 and CA3 using Nissl staining method (cresyl violet). Cell viability was verified based on the shape and strength of the stain (Figure 4a). Fluoro-Jade staining images presented to the right of cresyl violet staining images show cells undergoing neurodegeneration. Neurodegenerative cells were considered positive by their fluorescence and were quantified accordingly.

Cell viability in CA1 was significantly lower in the KA + Veh group than in the Veh + Veh group. Cell survival in CA1 of the KA + LE 1% group was significantly greater than that of the KA + Veh group, suggesting that LE provided neuroprotection (Figure 4b, left). In addition, neurodegeneration was significantly lower in the KA + LE 1% group compared to the KA + Veh group (Figure 4b, right). Although LE treatment provided neuroprotection in a dose-dependent manner, the KA + LE 0.01% group did not demonstrate a significant degree of protection. The consequential hippocampal cell death in the CA3 induced by seizures was not reduced by LE (Figure 4c). Protection of CA3 may have been deficient due to the injection time point of LE which occurred after 90 min of convulsive seizures. In a previous study, silencing synapses in CA3 attenuated the degree of seizures and reduced seizure-induced neuronal death in the CA3, but not CA1, region [36].

### 2.5. LE Activates Cell Survival Signals Involved in the Wnt Signaling Pathway

**Figure 5:**
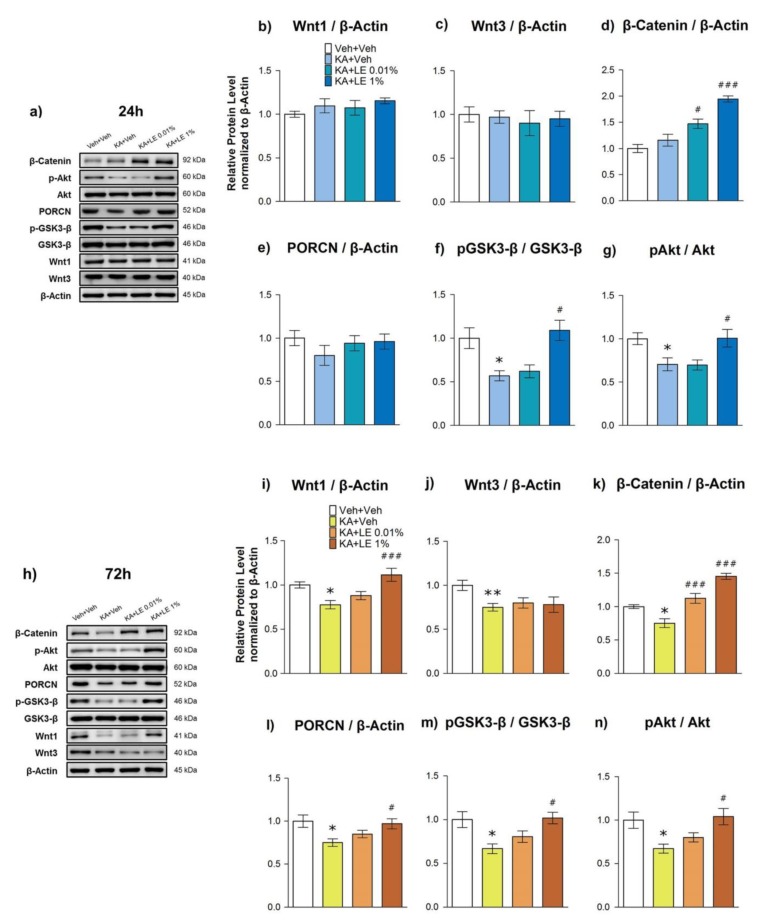
Effects of lipid emulsion on protein expression in hippocampi extracted 24 or 72 h after kainic acid (KA) and repetitive lipid emulsion (LE) injection (1 and 3 times, respectively). (**a**–**g**) Protein expression in hippocampi extracted 24 h after kainic acid injection. The KA + Veh group exhibited significant attenuation of p-Akt and p-GSK3-β. Wnt1, Wnt3, and PORCN did not exhibit any significant changes across all groups. Expression levels of β-catenin were increased significantly in the LE-injected groups, while those of p-Akt and p-GSK3-β were significantly increased only in the KA + LE1% group. (**h**–**n**) Protein expression in hippocampi extracted 72 h after kainic acid injection. The KA + Veh group exhibited significant attenuation of Wnt3, PORCN, p-Akt, and p-GSK3-β expressions. Wnt1, Wnt3, β-catenin, and PORCN expressions significantly decreased in the KA + Veh group compared to those in the Veh + Veh group. Expression levels of β-catenin increased significantly in the LE-injected groups, while those of PORCN, p-Akt, and p-GSK-3β significantly increased only in the KA + LE 1% group. Data are presented as mean ± standard error of the mean (SEM); *n* = 10 for each group; b-n) * *p* < 0.05 vs. Veh + Veh, # *p* < 0.05, ### *p* < 0.001 vs. KA + Veh, one-way analysis of variance (ANOVA) followed by Tukey’s multiple comparison test.

Although the mechanism of action of LE in the brain has not been clearly identified, a known protective mechanism in the heart mainly involves the phosphorylation of Akt [22] and GSK3-β [24], which in turn promote cell survival. In particular, the phosphorylation of GSK3-β through the canonical Wnt signaling pathway is known to inhibit the degradation of β-catenin [16]. Based on the increased levels of *Wnt1* mRNA expression at 24 h after KA administration in LE-treated groups, we examined the protein expressions at 24 h (Figure 5a) and 72 h (Figure 5h), and the phosphorylation of related protein markers: Wnt1, p-Akt/Akt, p-GSK3-β/GSK3-β, Porcupine (PORCN), and β-catenin.

In contrast to the *Wnt1* mRNA expression, there were no significant changes in the levels of Wnt1 protein expression at 24 h (Figure 5b), but differences were observed at 72 h after KA administration (Figure 5i). The change in Wnt1 expression levels at different time points (Supplementary Materials S1) was assumed by the decrease in neuronal populations in the CA1 region, as shown in Figure 4a,b, which in turn may have decreased Wnt1 expression in general. PORCN, an upstream marker known to activate Wnt signals [37], decreased significantly at 72h in the KA + Veh group (Figure 5l). Lower expression of PORCN in the KA + LE 0.01% group compared to the KA + LE 1% group relates to the decrease in Wnt1 expression at 72 h. Significant changes in downstream markers were consistently observed at both 24 h and 72 h after KA administration, indicating cell survival. The prevention of β-catenin degradation (Figure 5d,k) in LE-treated groups was related to increased phosphorylation activity in upstream GSK3-β signaling (Figure 5f,m), which is regulated by Wnt1. Akt (Figure 5g,n) protein expression levels in LE-treated groups also increased, which may have promoted cell survival. β-catenin protein expression levels were significantly upregulated in LE-treated groups at both time points. β-catenin protein expression was comparably lower at 72 h than at 24 h after KA administration (Supplementary Materials S1). The decrease in signal strength in all KA-treated groups may have been the result of cell death in the CA3 region induced by convulsive seizures, as shown in Figure 4c, which may have downregulated the signal in general due to reduced neuronal population size. Compared to the Veh + Veh group, Wnt3 was also downregulated in all experimental groups at both 24 h and 72 h (Figure 5c,j). There were no differences in the level of GSK3-β and Akt protein expression at 24 h and 72 h within groups (Supplementary Materials S1), indicating a persistent effect of survival signals through repetitive LE injections at the acute stage.

### 2.6. Significant mRNA Differences in Canonical Wnt Signaling

**Figure 6:**
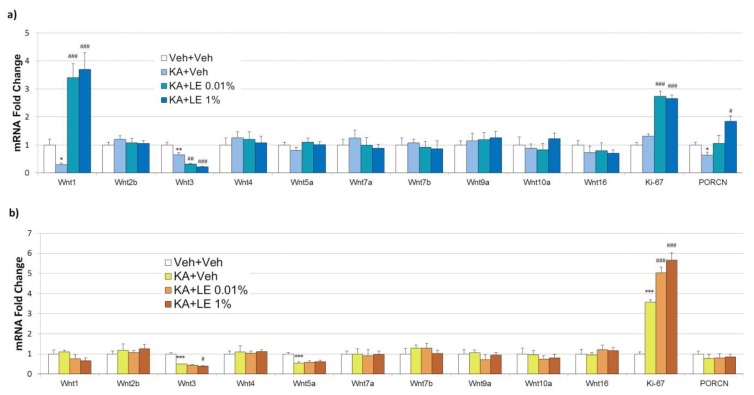
Effects of lipid emulsion on mRNA expression levels in hippocampi extracted 24 or 72 h after kainic acid (KA) and repetitive lipid emulsion (LE) injection (1 or 3 times, respectively). (**a**) mRNA expression levels for each experimental group at 24 h after kainic acid injection. Expression levels of Wnt1 and Ki-67 were significantly increased, while those of Wnt3 were attenuated in LE-injected groups. The expression level of PORCN increased significantly in the KA + LE 1% group compared to the KA + Veh group. (**b**) The mRNA expression levels for each experimental group at 72 h after kainic acid injection. Significantly lower levels of Wnt3 and Wnt5a expressions were observed. Significantly increased levels of Ki-67 were observed in all groups except the Veh + Veh sham group. Data are presented as mean ± standard error of the mean (SEM); *n* = 10 for each group; * *p* < 0.05, ** *p* < 0.01, *** *p* < 0.001 vs. Veh + Veh, # *p* < 0.05, ## *p* < 0.01, ### *p* < 0.001 vs. KA + Veh, one-way analysis of variance (ANOVA) followed by Tukey’s multiple comparison test.

The Wnt signaling pathway consists of 19 families, comprising *Wnt1* to *Wnt16*. Each Wnt family has different roles, which are broadly divided into two categories: The canonical and non-canonical pathways. Canonical Wnt pathway involves the GSK3-β destruction complex, where β-catenin is degraded by proteasomes through ubiquitination. Non-canonical Wnt pathway involves c-Jun N-terminal kinases (JNK) and Ca^2 + ^ -dependent mechanisms for cell adhesion and growth [38,39]. In this study, Wnt signals presented in the rodent hippocampus were selected by screening genes of the Wnt family (Figure 6a,b). *Wnt1* and *Wnt3* of the canonical Wnt pathway, and *Wnt5a* of the non-canonical Wnt pathway, were selected as potential biomarkers related to neuroprotection.

*Wnt1* and *Wnt3* have been reported to be involved in cell proliferation and survival via modulation of the GSK3-β complex [40,41]. Phosphorylation of the GSK3-β complex inhibits the phosphorylation of β-catenin, which promotes cell survival in the brain [42]. At 24 h after KA administration, a 10-fold difference in *Wnt1* expression between the KA + Veh group and LE-injected groups was observed (Figure 6b), indicating significant activation of the Wnt/β-catenin pathway. The level of *Wnt1* mRNA expression in the KA + Veh group was significantly lower than that in the Veh + Veh group, implying attenuated levels of cell survival. *Wnt1* expression substantially exceeded the baseline (represented by the Veh + Veh group), implying that overexpression of *Wnt1* underpinned neuroprotection in pathological conditions (Figure 6b). Despite a peak in activities of *Wnt1* at the 24 h time point, it returned to baseline or lower in LE-injected groups at 72 h after KA injection. The attenuation in *Wnt1* at 72 h was notable but not significant due to high levels of variability in each group. The expression of the upstream signal, PORCN was significantly increased in the 24 h group but not in the 72 h group, indicating the activation of Wnt at the acute phase of the pathological state. Expression of another canonical Wnt signal, *Wnt3* was more dramatically attenuated in the LE-treated groups compared to the difference in expression between the KA + Veh and Veh + Veh groups at 24 h after KA administration. Lower expression levels persisted until 72 h after KA administration, indicating a reduction in aberrant neurogenesis, which is a therapeutic goal in excitotoxic conditions.

*Wnt5a* is a non-canonical Wnt signal that activates the JNK and Ca^2 + ^ pathways for dendritic maintenance and post-synaptic assembly of glutamate receptors [43]. There were no significant differences in *Wnt5a* mRNA expression between groups at 24 h, but a significant decrease was observed at 72 h after KA administration in all KA-treated groups compared to Veh + Veh. This implied the absence of interaction between LE and Wnt5a in pathological conditions induced by KA. Increased levels of *Ki-67*, a cell proliferation marker that is also influenced directly by the canonical Wnt signaling pathway [44], was measured in LE-treated groups at 24 h after KA administration. Subsequently, all groups excluding the Veh + Veh group exhibited elevated expression levels of *Ki-67* at 72 h after KA administration. This increased expression may have been underscored by multiple factors such as the aberrant neurogenesis of glial cells in pathological conditions induced by KA at particular time points.

## 3. Discussion

The results of our study suggested that LE provided neuroprotection against acute excitotoxic neural damage in a dose-dependent manner. Compared to lower concentrations, the concentration of LE 1% was the most effective at providing neuroprotection in the hippocampus. The administration of LE also lowered mortality induced by KA nearly to a significant level (Figure 1). Many animals of the KA + Veh group experienced difficulty in maintaining a healthy status. On the other hand, stabilized behavior was observed in most animals of the KA + LE 1% group, which may be due to the partial alleviation of neurotoxicity. Lower concentrations were less effective than the higher dose when administered locally. A previous study reported that intraperitoneal injections of higher doses did not seem to alter brain state [45], but differed in intrahippocampal injections from our study. The level of *Wnt1* mRNA expression was up-regulated at 24 h after KA + LE injection, but this returned to baseline or lower at 72 h after KA + LE injection. These data suggest that LE triggers neuroprotection at the acute phase of excitotoxicity.

Memory and anxiety levels were protected in the LE 1% treated group when compared to the LE 0.01% treated group. Despite the loss of neurons in the CA3 region, protection in CA1 was sufficient to secure hippocampal function. However, it is important to note that the behavioral tests were not heavily influenced by CA3 impairment. The CA3 region is involved in the processing of spatial memory [46] and is affected by convulsive seizures accompanied by KA administration [28,34]. A previous study reported that synaptic silencing of the CA3 region significantly reduced seizures and cell death in that particular region [36]. Although LE does not possess known tranquilizing components for relieving seizures, accumulating evidence suggests that LE aids the survival of neurons in excitotoxic conditions. In a previous study, the protective effects of LE and propofol were measured by intracerebroventricular microinjections into the brains of rats with ischemia. LE significantly reduced the level of extracellular glutamate in the CA1 region during ischemia [47], suggesting that LE may be an important factor against excitotoxicity. Consequently, we observed that hippocampal cells in the CA1 region were significantly protected by LE. The attenuation of anxiety-like behavior, known to involve CA1 [48,49], was also consistent with the dose-dependent protective effects of LE in our behavioral tests. These findings indicate that LE provides neuroprotection in the hippocampus, but these effects exclude the damage induced by seizures.

The Wnt signaling pathway has received attention as a therapeutic target for neurodegenerative diseases across numerous studies based on upregulated antagonistic activity in pathological conditions [50,51]. Promoting Wnt activation through the inhibition of antagonists resulted in significant recovery from pathological conditions [17,52]. Wnt1 is an upstream signal of GSK3-β that was up-regulated in LE-treated groups, supporting cell survival through the regulation of β-catenin. The preservation of β-catenin through the phosphorylation of GSK3-β is often reported as a consequence of canonical Wnt signaling [16]. The effects of LE on GSK3-β and Akt have not been clearly elucidated, but the elevated levels of these signals indicate an interaction induced by the constituents of LE. One possible explanation for the initiation of Wnt may involve the lipid modification of Wnt1. Wnts are glycoproteins that are modified by glycosylation and lipids through palmitoylation partly by PORCN. Such modifications can trigger cell survival mechanisms in the pathological state of excitotoxicity. Protective effects of LE in pathological conditions increased *Wnt1* mRNA expression levels according to our findings. The downstream protection-related protein markers, Akt and GSK3-β, was consistent with previous studies in myocardial cells [24,53]. Although metabolic factors differed between neurons and cardiomyocytes, it is notable that survival signals were activated regardless. In addition, Wnt3 was attenuated in LE-treated groups, implying that not all canonical Wnts displayed uniform trends in the levels of expression for neuroprotection against excitotoxicity. Canonical Wnt3 signal has been reported to be associated with neurogenesis in the hippocampus [41]. The expression of Wnt3 may have persisted at a low level in the present study, as KA has been reported to encompass aberrant neurogenesis in the hippocampus at chronic but not acute phases of its pathology [54]. A possible explanation for the significantly lower expression levels of Wnt3 in LE-treated groups may be related to the survival of CA1 neurons. Protection provided to CA1 neuronal populations may have affected inflammatory responses that triggered gliosis surrounding the damaged area. On the other hand, the non-canonical Wnt5a has been associated with the configuration of postsynaptic compartments and cellular structures [43,55]. The difference in Wnt5a expression was insignificant between KA + Veh and LE-treated groups, indicating minimal differences in structures affected by non-canonical signaling.

Although significant neuroprotection was observed through the outcomes of the present study, there are some limitations. Our study focused on the local effects of LE in the brain and observed neuroprotective properties by direct injection. Moreover, the systemic effects of LE in the brain are at question. The local injection of LE in clinical settings may lack practicality before it has been compared with other routes of administration. Intravenous or intracarotid injections are possible routes that can examine the systemic effects of LE. Dosage screening would need to be accompanied for different routes of administration for the search of effective concentrations. In addition, cell death was observed in higher concentrations of LE in our dosage screen using organotypic hippocampal slice cultures (Supplementary Materials S2). The exposure to excess amounts of LE may have adverse effects on the brain. Therefore, the search for appropriate doses for different routes of administration is necessary in future studies. 

In conclusion, LE administration resulted in alleviation of hippocampal damage in the acute phase of excitotoxicity induced by KA. Our results were consistent with previous studies regarding the phosphorylation of Akt [22] and GSK3-β [24] through LE administration in the heart. Furthermore, we validated the aforementioned survival markers as part of the canonical Wnt signaling pathway. In particular, *Wnt1* mRNA expression levels were significantly increased, and β-catenin was preserved from degradation, thereby promoting the survival of hippocampal cells under excitotoxic conditions. Neuroprotection provided by LE significantly reduced the exacerbation of cognitive function and anxiety induced by KA through the protection of the hippocampal CA1 region. These findings suggest that LE is neuroprotective in KA-induced excitotoxicity in the hippocampus in vivo and provide a foundation for further investigation of therapies against excitotoxic neural injuries. 

## 4. Materials and Methods

### 4.1. Animals

Adult Sprague Dawley rats weighing 200–250 g (Koatec, Pyeongtaek, South Korea) were used for the experiment in this study. Animals were housed in groups of 3 per cage under 12 h light/dark cycles, with free access to food and water. Animals were subjected to 7 days of acclimation upon arrival at the Association and Accreditation of Laboratory Animal Care (AAALAC)-accredited Yonsei University College of Medicine Animal Care Facilities. All experimental procedures were performed according to the National Institutes of Health Guide for Care, and were approved by the Institutional Animal Care and Use Committee of Yonsei University Health System (permit no.: 2016-0100, approval date: 7 December 2016).

### 4.2. Stereotaxic Surgery and Cannula Implantation

**Figure 7:**
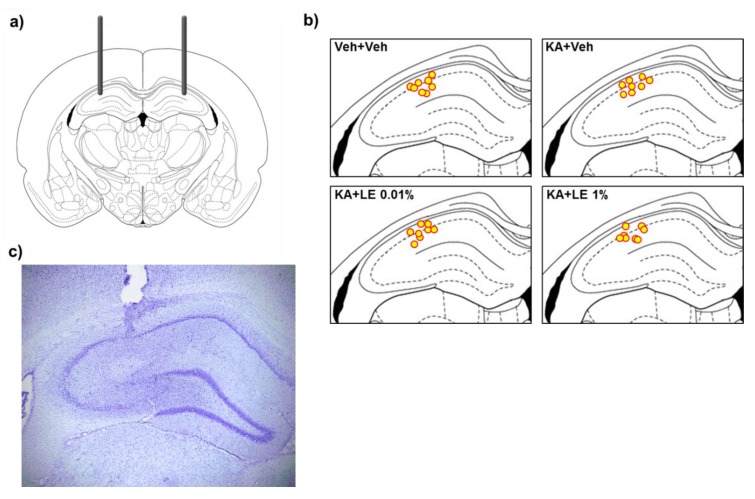
Illustration of stereotaxic implantation of guide cannulas. (**a**) Bilateral implantation of guide cannulas into the CA1 region of the rat hippocampus. (**b**) Localization of injection sites after stereotaxic implantation. (**c**) Site of injection visualized in a cresyl violet-stained section. (**a**–**b**) Drawings of the rat brain have been traced from Paxinos and Watson [56].

Rats were anesthetized by intraperitoneal (i.p.) injection of 50 mg/kg sodium pentobarbital (Hanlim Pharmaceutical, Seoul, South Korea) and were placed in a stereotaxic frame (David Kopf Instruments, Tujunga, CA, USA) for surgical procedures. Stainless steel guide cannulas (22-gauge, 4 mm long, Plastics One, Roanoke, CA, USA) were bilaterally implanted into the CA1 region of the hippocampus (from bregma: Anterior/posterior, −3.3 mm; medial/lateral, ±2.4 mm; dorsal/ventral, −3.0 mm). Cannulas were secured with dental acrylic cement with stainless steel screw anchors fixed to the skull. Obturators were placed in the guide cannulas after surgery, and rats were returned to their home cages for 1 week of recovery. Injection sites are illustrated in Figure 1b and Figure 7a and were verified through cresyl violet staining (Figure 7c).

### 4.3. Drug Treatment

KA (K0250, Sigma Aldrich, St. Louis, MO, USA) was dissolved in sterile 0.9% NaCl to a final concentration of 0.8 μg/μL. KA was microinjected bilaterally into the hippocampus through internal cannulas (26-gauge, 5 mm long, Plastics One) at a volume of 1 μL over 1 min. The injectors remained in place for an additional 1 min before being replaced by obturators. Lipid emulsion was dissolved in sterile 0.9% NaCl to final concentrations of 0.01% and 1% lipid emulsion (LE). The potential doses of LE were selected from a pilot study in vitro using organotypic hippocampal slice cultures (Supplementary Materials S2). The vehicle, LE 0.01%, and LE 1% were intrahippocampally administered 90 min after KA injection via internal cannulas at a rate of 1 μL over 1 min. Additional injections of vehicle, LE 0.01%, and LE 1% were administered repetitively 24 h and 48 h after the initial KA injection time points for the experimental groups sacrificed at 72 h. Seizures were terminated by intramuscular injection of 10 mg/kg diazepam (Dong Wha Pharmaceutical, Seoul, South Korea) 90 min after the initial KA injection. Only rats that experienced a seizure level of 3 (forelimb clonus), 4 (rearing), or 5 (falling after rearing) of the Racine scale [57,58] were included.

### 4.4. Passive Avoidance Test

Passive avoidance test was conducted using Gemini™ (San Diego Instruments, San Diego, CA, USA) in a dark room. Three days after the administration of KA, behavioral tests commenced with a habituation period in which rats were allowed to freely explore the apparatus for 5 min. On the next day (acquisition trial), animals were habituated to the testing room for 2 h before the behavioral test. The chambers were illuminated, and stepover time was recorded. Rats were then subjected to 1.5 mA electrical footshock for 3 sec (Figure 2a). Rats were returned to their cages 5 sec after the footshock. Rats that did not step over to the darker chamber after 90 sec were not considered for this behavioral test. Twenty-four hours after the acquisition trial, stepover time for retention was recorded for a maximum of 90 sec. Rats were subjected to identical conditions from the training session but without the delivery of the electrical footshock. The entire recording process of stepover latencies was recorded automatically using Gemini™ software (GEMINI Avoidance System, San Diego Instruments,).

### 4.5. Elevated Plus Maze

Elevated plus maze (EPM) consisted of 2 open arms and 2 closed arms (Figure 3a). Each arm was 50 cm in length, 10 cm wide, and 50 cm elevated from the ground; while closed arms were enclosed with 40 cm opaque walls. The EPM test was conducted 4 days after KA injection for all experimental groups. Rats were left in the testing room for 2 h of habituation prior to actual trials on the apparatus. The experimental animals were then placed in the center platform of the EPM facing an open arm. Rats were free to explore the apparatus for 5 min while the duration and number of entries of either the open or closed arms were measured. Valid entries to either the closed or open arms were considered when all 4 paws of the rat entered the arm.

### 4.6. Sample Preparation

Hippocampal tissues were collected 24 h or 72 h after KA injection for each group. The experimental animals were anesthetized with isoflurane and decapitated for tissue collection. Tissue samples were immersed in liquid nitrogen immediately after extraction. For half of the experimental groups, the left hippocampus was used for qPCR analysis, and the right hippocampus was used for immunoblotting analysis. For the other half of the experimental groups, treatment was counterbalanced (left side for immunoblotting and right side for qPCR) in tissue extraction to minimize animals sacrifice. Frozen tissues were used for either qPCR or immunoblotting within 24 h upon tissue extraction.

### 4.7. Histological Staining

Animals were deeply anesthetized with urethane (1.25 g/kg, i.p.) at 24 h or 72 h after KA injection for each experimental group and transcardially perfused with 0.9% NaCl via the ascending aorta followed by perfusion with 4% paraformaldehyde (0.1 M phosphate buffer, pH 7.4). Extracted brain tissues were immersed in the same 4% paraformaldehyde solution for 24 h at 4 °C. After post-fixation, tissues were stored in 10% to 30% sucrose gradient (0.1 M phosphate buffered saline, pH 7.4) for cryoprotection. Brain tissues were instantly frozen at −60 °C in cooled isopentane and sectioned onto silane-coated slides at a thickness of 20 μm.

Histological staining was conducted by washing slides in 0.1 M phosphate buffered saline (PBS) and staining in 0.25% cresyl violet acetate (C5042, Sigma Aldrich,) for 2 min. The slides were rinsed in tap water for 1 min and immersed into 70%, 95%, and 99.9% ethanol for 10 s each. Then, the slides were placed in xylene for dehydration and mounted with coverslips using Permount (SP15, Fisher Scientific, Fair Lawn, NJ, USA).

Neurodegenerative staining was conducted by immersing slides into a basic alcohol solution consisting of 1% sodium hydroxide in 80% ethanol for 5 min. The slides were then rinsed in 70% ethanol and distilled water for 2 min each. Slides were incubated in 0.1% potassium permanganate solution for 30 min. Following a 2 min wash with distilled water, the slides were transferred for 2 h to 0.0001% solution of Fluoro-Jade C (AG325, Merck Millipore, Temecula, CA, USA) dissolved in 0.1% acetic acid. Slides were then rinsed 3 times in distilled water and coverslipped using DPX mounting solution (06522, Sigma Aldrich).

### 4.8. Western Blotting

Frozen samples were homogenized in lysis buffer (ProPrep; Intron Biotechnology, Pyeongtaek, South Korea) with phosphatase inhibitors (Phosstop; Roche, Mannheim, Germany) for protein extraction. Supernatants were collected from homogenized samples that were centrifuged at 15,000 rpm for 15 min at 4 °C. Total protein concentrations were measured using a spectrophotometer (Nano Drop ND-1000, NanoDrop Technologies Inc., Wilmington, DE, USA), and proteins of equal amounts (30 mg per well) were inserted for denaturation at 94 °C. Samples were then loaded on 10% SDS-PAGE and transferred to a polyvinylidene difluoride membrane (Merck Millipore, Darmstadt, Germany) for over 2 h. Phospho-proteins were immunodetected prior to the corresponding total protein after the membrane had been stripped. Transferred proteins on membranes were fixed using 0.05% glutaraldehyde in TBS-0.05% Tween-20 (TBST) for 15 min at room temperature. The membrane was stained with Ponceau S (Sigma-Aldrich) for visualization of the transferred proteins and cut into strips according to the size of the target protein to minimize interactions between antibodies. Membranes were blocked with 5% bovine serum albumin (BSA) dissolved in Tris-buffered saline with Tween 20 (TBST) for 1 h at room temperature. The membranes were incubated overnight with primary antibodies diluted in 5% BSA in TBST at 4 °C. Rabbit was the host of all primary antibodies used for immunoblotting. The following antibodies were used: Anti-Wnt1 (ab15251, 1:1000, Abcam, Cambridge, UK), anti-β-Catenin (#9562, 1:3000, Cell Signaling Technology, Beverly, MA, USA), anti-Akt (#4691, 1:3000, Cell Signaling Technology), anti-Phospho-Akt (#9271, 1:1000, Cell Signaling Technology), anti-GSK3-β (#9315, 1:3000, Cell Signaling Technology,), anti-Phospho-GSK3-β (#9336, 1:1000, Cell Signaling Technology,), and anti-β-Actin (#4967, 1:10,000, Cell Signaling Technology,). After overnight incubation of primary antibodies, the membranes were immersed with anti-rabbit horseradish peroxidase-conjugated secondary antibody (#7074, 1:10,000, Cell Signaling Technology,). Visualization of immunoreactive proteins was performed with the application of chemiluminescent detection reagent (ECL™ Prime, GE Healthcare, Little Chalfont, UK) and by ImageQuant™ LAS 4000 (GE Healthcare). Protein immunoreactivity was measured using Multi Gauge software (Multi Gauge V3.0, Fuji Film Inc., Tokyo, Japan). 

### 4.9. qPCR

Hippocampal tissue RNA extraction was executed using the Hybrid-R kit (305-010; GeneAll Biotechnology, Seoul, South Korea). RNA concentration was measured using a spectrophotometer (Nano Drop ND-1000, NanoDrop Technologies Inc.). cDNA was prepared from 1 μg of total RNA using the PrimeScript 1st strand cDNA synthesis kit (Takara Bio, Shiga, Japan). PCR amplification was executed using the SYBR-Green reagent (Takara Bio) in the ABI 7500 real-time PCR system (Applied Biosystems, Foster City, CA, USA). PCR amplification was performed in 20 μL reaction volumes. Sequences for oligonucleotide primers were selected using the Gene Database of National Center for Biotechnology Information (NCBI) and Primer Express™ Software v3.0.1 (Thermo Fisher Scientific, Waltham, MA, USA). Primer pairs are listed in Table 3 and were verified using a melting curve analysis (Supplementary Materials S3).

### 4.10. Statistical Analysis

Statistical evaluations were performed using one-way ANOVA or unpaired *t*-test, as indicated in figure legends. Post-hoc analyses were performed using the Tukey’s multiple comparisons test or as otherwise specified in the figure legends. All statistical analyses were performed using GraphPad Prism software (5.03 GraphPad Software Inc., San Diego, CA, USA). A *p*-value less than 0.05 was considered statistically significant for all analyses.

## Figures and Tables

**Table 1:** Seizure severity of experimental animals measured using Racine’s scale. A total of 220 animals were assessed for their seizure behavior and scaled accordingly to their behavior. The Veh + Veh group were not included in this table because they were not administered with KA and did not experience seizures.

Racine’s Scale Stage/Class	Behavior	% of Total Exp. Animals
1	Immobility, orofacial movements	0% (0/220)
2	Head nodding, facial clonus	17% (37/220)
3	Forelimb clonus, wet dog shakes	68% (150/220)
4	Forelimb clonus with rearing	9% (20/220)
5	Clonic rearing and falling, wild jumping	6% (13/220)

**Table 2:** Number at risk and survival rate by time. Total of 260 animals (*n* = 65 per group) were assessed on the survival after the injection of vehicle or KA.

**Number at Risk by Time**				
	Day 0	Day 1	Day 2	Day 3
Veh + Veh	65	65	65	65
KA + Veh	65	58	52	47
KA + LE0.01%	65	63	55	51
KA + LE1%	65	65	62	58
**Survival Rate by Time**				
	Day 0	Day 1	Day 2	Day 3
Veh + Veh	1	1	1	1
KA + Veh	1	0.892	0.800	0.723
KA + LE0.01%	1	0.969	0.846	0.785
KA + LE1%	1	1	0.954	0.892

**Table 3:** Primer pairs for qPCR.

Gene name	Forward Primer (5′-3′)	Reverse Primer (5′-3′)
Wnt1	*GCAACCAAAGTCGCCAGAA*	*TATGTTCACGATGCCCCACCA*
Wnt2b	*GCTACCCAGACATCATGCG*	*ACACTCTCGGATCCATTCCC*
Wnt3	*AATTTGGTGGTCCCTGGC*	*GATAGAGCCGCAGAGCAGAG*
Wnt4	*GTTTCCAGTGGTCAGGATGC*	*AGGACTGTGAGAAGGCTACGC*
Wnt5a	*AAGGGAACGAATCCACGCC*	*ATACTGTCCTGCGACCTGCTTC*
Wnt7a	*CCAAGGTCTTCGTGGATGC*	*TGTAAGTTCATGAGGGTTCGG*
Wnt7b	*CGTGTTTCTCTGCTTTGGC*	*CACCACGGATGACAATGC*
Wnt9a	*GTACAGCAGCAAGTTTGTCAAGG*	*CACGAGGTTGTTGTGGAAGTCC*
Wnt10a	*CGGAACAAAGTCCCCTACG*	*AGGCGAAAGCACTCTCTCG*
Wnt16	*GCACTCTGTAACCAGGTCATGC*	*TGCAAGGTGGTGTCACAGG*
Ki-67	*TTCAGTTCCGCCAATCCAAC*	*CCGTGCTGGTTCCTTTCCA*
PORCN	*CCTACCTCTTCCCCTACTTCA*	*CTTTGCGTTTCTTGTTGCGA*
β-Actin	*GTCCACCCGCGAGTACAAC*	*TATCGTCATCCATGGCGAACTGG*

## Supplementary Materials

Supplementary materials can be found at https://www.mdpi.com/1422-0067/21/8/2706/s1.

## Abbreviations

AAALAC	Association and Accreditation of Laboratory Animal Care
Akt	Protein kinase B
ANOVA	Analysis of variance
CA	Cornu Ammonis
CNS	Central nervous system
Dkk-1	Dickkopf-related protein 1
FJC	Fluorojade-C
GSK3-β	Glycogen synthase kinase-3β
JNK	c-Jun N-terminal kinases
KA	Kainic acid
LAST	Local anesthetic systemic toxicity
LE	Lipid emulsion
mRNA	Messenger ribonucleic acid
PORCN	Porcupine
qPCR	Quantitative polymerase chain reaction
SEM	Standard error of mean
Veh	Vehicle
Wnt	Wingless integration
