# Supplementary material for: Neuroprotection from Excitotoxic Injury by Local Administration of Lipid Emulsion into the Brain of Rats
Source: Int J Mol Sci. 2020 Apr 14;21(8):2706. doi: 10.3390/ijms21082706 (PMC7215821; doi:10.3390/ijms21082706)
Supplement: Supplementary file 1 [file ijms-21-02706-s001.pdf]

International Journal of *Molecular Sciences*

MDPI

Supplementary Materials

Neuroprotection from excitotoxic injury by local administration of lipid emulsion into the brain of rats

Motomasa Tanioka [1,2], Wyun Kon Park [3], Insop Shim [4], Kyeongmin Kim [1,2], Songyeon Choi [1,2], Un Jeng Kim [1], Kyung Hee Lee [5], Seong-Karp Hong [6], and Bae Hwan Lee [1,2,*]

[1] Department of Physiology, Yonsei University College of Medicine, Seoul 03722, Republic of Korea; hpark@yuhs.ac (M.T.); kkm0814@yuhs.ac (K.K.); sychoi@yuhs.ac (S.C.); mignon@yuhs.ac (U.J.K.)
[2] Brain Korea 21 PLUS Project for Medical Science, Yonsei University College of Medicine, Seoul 03722, Republic of Korea
[3] Department of Anesthesiology and Pain Medicine, Anesthesia and Pain Research Institute, Yonsei University College of Medicine, Seoul 03722, Republic of Korea; wkp7ark@yuhs.ac
[4] Department of Physiology, School of Medicine, Kyung Hee University, Seoul 02447, Republic of Korea; ishim@khu.ac.kr
[5] Department of Dental Hygiene, Division of Health Science, Dongseo University, Busan 47011, Republic of Korea; kyhee@gdsu.dongseo.ac.kr
[6] Division of Biomedical Engineering, Mokwon University, Daejeon 35349, Republic of Korea; karp@yuhs.ac
* Correspondence: bhlee@yuhs.ac; Tel.: +82-2-2228-1711

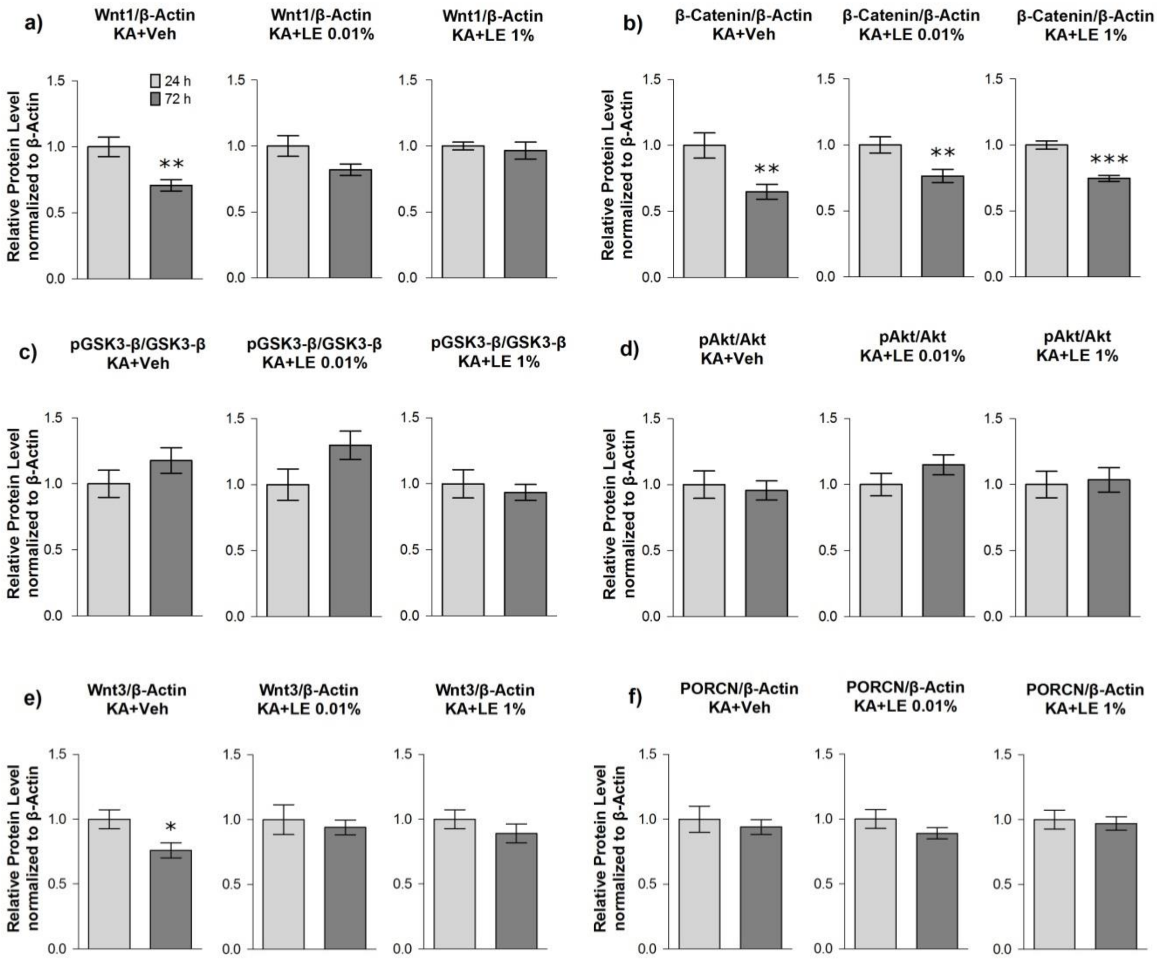

Supplementary Material S1. Comparison of lipid emulsion on protein expression between 24 h and 72 h after KA (kainic acid) injection. (a) Differences in Wnt1 protein levels between 24 h and 72 h time-points. There was a significant decrease in Wnt1 protein levels at 72 h compared to those at 24 h. (b) Differences in β-catenin protein levels between 24 h and 72 h time-points. There were significant decreases in all KA-injected groups. (c) Differences in p-GSK3-β/GSK3-β protein levels between 24 h and 72 h timepoints. (d) Differences in p-Akt/Akt protein levels between 24 h and 72 h timepoints. (e) Differences in Wnt3 protein levels between 24 h and 72 h timepoints. There was a significant decrease in Wnt3 protein expression in the KA+Veh group. (f) Differences in PORCN protein levels between 24 h and 72 h timepoints. There were no significant differences in the Veh+Veh group at 24 h and 72 h. Data are presented as mean ± standard error of mean (SEM); n=10 for each group; a-f) $^{**}P < 0.01$, $^{***}P < 0.001$ vs 24 h, unpaired *t*-test.

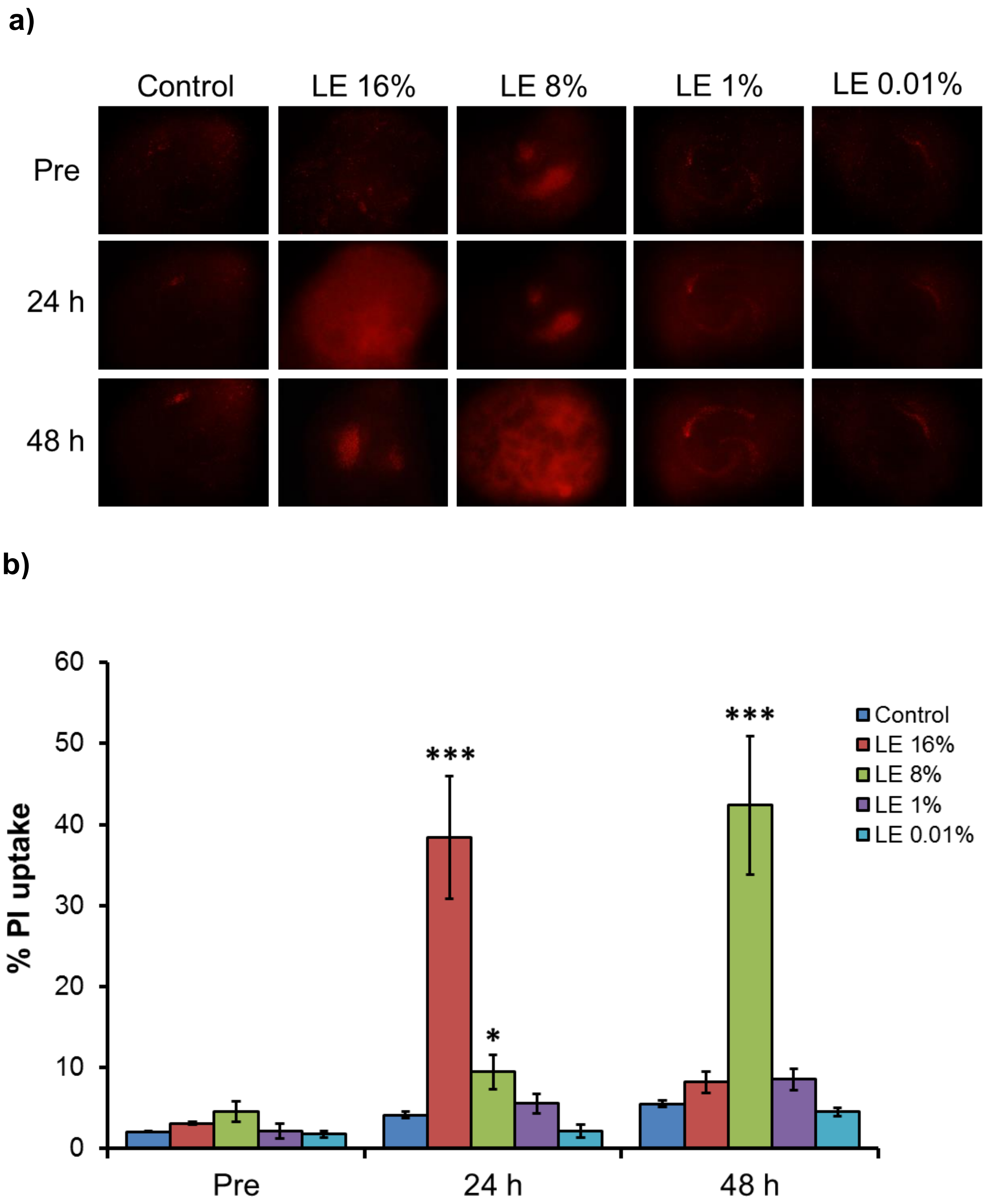

Supplementary Material S2. Propidium iodide staining of kainic acid-treated organotypic hippocampal slice cultures. (**a**) Propidium iodide staining of organotypic hippocampal slice cultures. Cultures were treated with either saline or different doses of lipid emulsion for up to 48 h. Positive signals indicate the occurrence of neuronal death. (**b**) Neuronal death occurred the most in LE 16% group at 24 h and in LE 8% group at 48 h. Such results indicate precautions in the selection of doses. Data are presented as mean ± standard error of mean (SEM); n=6 for each group; *P < 0.05, ***P < 0.001 vs Control; one-way ANOVA followed by Dunnett's post-hoc analysis for comparison with the Control (saline) group at each time period.

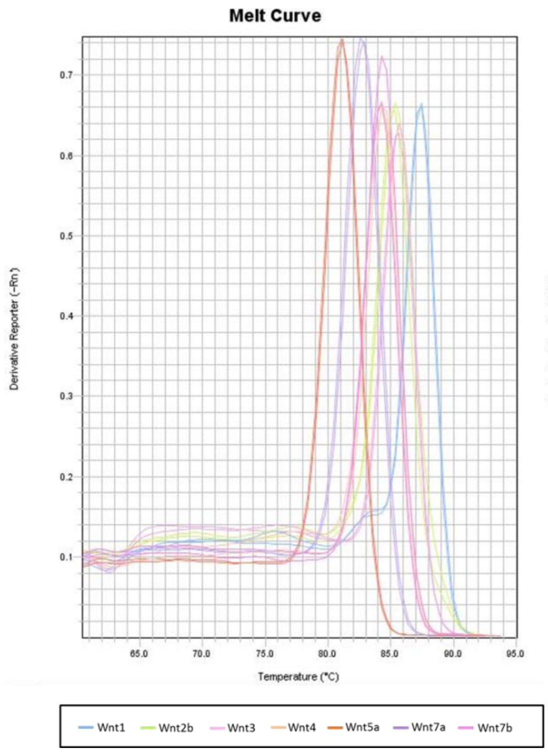

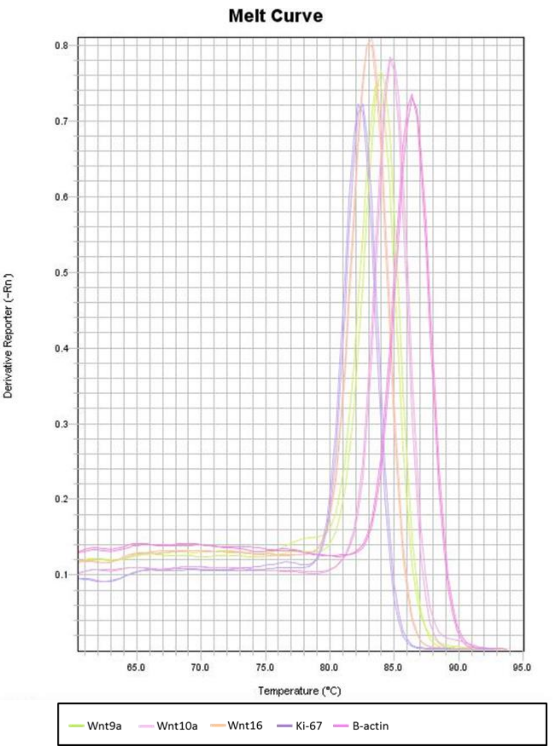

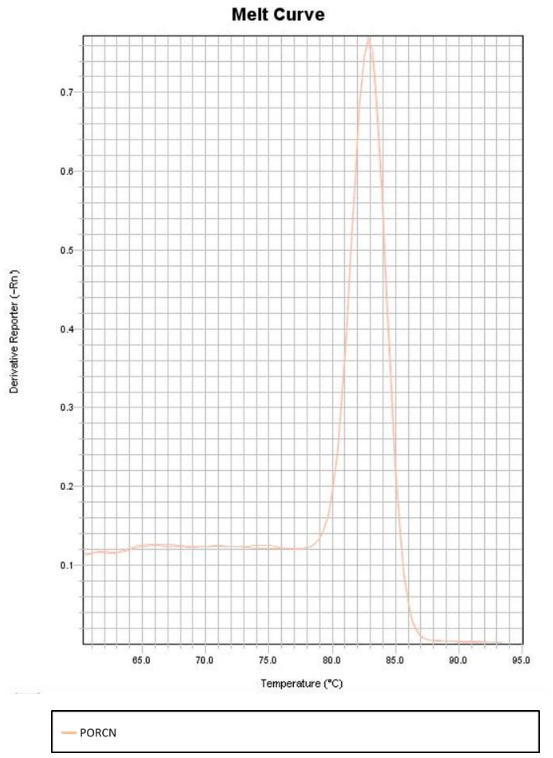

Supplementary Material S3. Melting curve of qPCR primers. Primers used for qPCR were verified by analyzing the melting point. A single peak for each target provided notable indications for effective binding specificity for primers.

© 2020 by the authors. Submitted for possible open access publication under the terms and conditions of the Creative Commons Attribution (CC BY) license (http://creativecommons.org/licenses/by/4.0/).
